# Laser-Based Trespassing Prediction in Restrictive Environments: A Linear Approach

**DOI:** 10.3390/s120911870

**Published:** 2012-08-29

**Authors:** Fernando Auat Cheein, Gustavo Scaglia

**Affiliations:** 1 Department of Electronics Engineering, Federico Santa Maria Technical University, Av Espana 1680, Valparaiso, Chile; 2 Chemistry Institute, San Juan National University, Av. San Martin 1109, San Juan, Argentina; E-Mail: gscaglia@unsj.edu.ar

**Keywords:** target tracking, linear prediction, range laser sensor

## Abstract

Stationary range laser sensors for intruder monitoring, restricted space violation detections and workspace determination are extensively used in risky environments. In this work we present a linear based approach for predicting the presence of moving agents before they trespass a laser-based restricted space. Our approach is based on the Taylor's series expansion of the detected objects' movements. The latter makes our proposal suitable for embedded applications. In the experimental results (carried out in different scenarios) presented herein, our proposal shows 100% of effectiveness in predicting trespassing situations. Several implementation results and statistics analysis showing the performance of our proposal are included in this work.

## 1. Introduction

The tracking and prediction of objects or targets has several applications, such as traffic surveillance [1], pedestrian detection [1,2], mobile robot autonomous navigation in dynamic environments [3,4], intelligent transportation systems [2,5], among others. Several of these applications require 2D and 3D target tracking, depending mainly on the number of degrees of freedom to be tracked by the system. Also, according to the application, the system can be focused on single and multiple targets tracking.

In general, a target tracking process can be divided into two main stages: *targets' detection* and *tracking procedure* [6]. The *targets' detection* stage is strongly related to the nature of the sensor used according to the application requirements. A wide range of sensors are currently used in objects or target tracking, such as artificial vision sensors and range laser sensors. With this insight, [7] uses a stereoscopic camera for visual tracking of 3D objects; [8] uses a video sequence for single object tracking, whereas [9] uses a monocular vision system for rigid single object tracking; also, [10] presents a monocular vision system for object tracking of moving objects, although the authors implement their system on a mobile robot for following purposes. In [6], the authors use video frames for multiple objects tracking, whereas [11] also uses video frames but for single object tracking.

Several procedures are used for object detection in artificial vision based applications. In [7], the authors use the FFT (Fast Fourier Transform) of the image to detect a dark object over a white background; a similar approach is presented in [1], where the Fourier transform is used to extract features from a video sequence for surveillance applications. In [12], the authors use frame differentiation and adaptive background subtraction combined with simple data association techniques to extract features. For multi-object tracking, [6] uses a spatio-temporal segmentation for features extraction from images. In [13] the authors present an online EM-algorithm for visual estimation of objects' parameters. The former are examples of objects' tracking and detection using artificial vision systems.

Range laser sensors are also used for target tracking applications, such as the case shown in [14], where a range laser sensor is used for environment modeling when applying a SLAM (Simultaneous Localization and Mapping) algorithm. A SLAM algorithm is used in mobile robot applications [3,4,15–18] to concurrently estimate the robot's position within an environment and to build a model of such an environment. The latter is accomplished by using exteroceptive sensors, such as range lasers, vision systems, ultrasonic sensors, *etc.* The model built of the environment usually contains the static and dynamic—or moving—elements. Such moving elements are tracked using the same estimation algorithm implemented for the SLAM execution—such as a Kalman Filter, and Information Filter, a Particle Filter, and their respective extensions (see [16,19–21] for further information). The object detection is related to the model of the environment. Thus, in [3,4], lines and corners are used for objects determination.

In addition, range laser sensors are also used for intruders detection, trespassing situations and workspace determination, as pointed out by the manufacturers [22,23]. However, it is worth mentioning that such applications are static: the workspace and the sensors' positions remain unchanged during the implementation and execution of the system. The intruders detection is based on a threshold determination: if the intruder trespass the protected workspace, a previously determined action is performed, regardless the intention of the intruder. Such an application is usually used in surveillance systems and workspace protection in factories [23].

Despite the detection algorithm and the sensor used by the system, the *tracking procedure* problem can be solved by several approaches (in this work, we consider the prediction problem as an extension of the tracking problem *per se*). Thus, [24] uses neural networks for multiple object tracking; [9] uses a Kalman Filter for real time tracking; [11] uses an adaptive block matching for the estimation of single object's motion. In [25], the authors propose a passive monitoring system based on a Gaussian model of the motion of the object; [2] uses the Bhattacharyya coefficient for visual tracking and [26] uses the Particle Filter as a tracking algorithm. However, [27] uses a star algorithm for visual tracking. Considering that prediction is possible by means of an appropriate tracking strategy, several approaches can be found with this scope. Thus, in [28] the authors propose a tracking and predicting approach based on the *AdaBoost* algorithm for multiple pedestrian scenarios; in [29], the authors present a particle filtering approach for predicting car's motion. On the other hand, [30] presents the tracking performed by the Extended Kalman Filter for predicting mobile robot's motion. As can be seen, several approaches can be used to solve the tracking and prediction problem, such as empirical procedures, user dependent decisions and estimation algorithms.

The Taylor's series expansion is also used as a tool for the object tracking and prediction problem. In [2] the Taylor's expansion is used to obtain a linear model of the Bhattacharyya coefficient used in the prediction procedure; [9] uses the Taylor's expansion for linearization of the motion model in the Kalman Filter. In [13], the Taylor's series expansion is used for the linearization of the objective function of the optical flow used in the target tracking application. As can be seen, the Taylor's series expansion is used for linearization purposes of intermediate process within the main tracking procedure. A more extended introduction and state of the art in target tracking procedures can be found in [31–34].

The main contribution of this work is a workspace supervision application based on the prediction of trespassing situations by using multiple stationary range laser sensors. The last is accomplished by using the Taylor's series expansion of the motion of the detected targets as a tracking—and predicting— procedure *per se*. Despite the fact that our method is implemented using range laser sensors, the Taylor's series expansion as a tracking procedure proposed in this work is independent of the nature of the sensor. In addition, the Taylor's series expansion as a tracking procedure allows us to predict the trespassing risks before they occur. We have also implemented our proposal for multi-targets prediction. For each proposed situation—single laser with single target, multiple lasers with single target, single laser with multiple targets and multiple lasers with multiple targets—we have performed real time experimentation and statistical analysis showing the advantages of our proposal.

This work is organized as follows: Section 2 shows an overview of the proposed system, the sensors description, the problem's hypothesis and the mathematical formulation of the proposal; Section 3 shows the experimentation and statistical results of each proposed situation. Section 4 presents the pros and cons observed during the experimentation stage. Section 5 shows the conclusions of this work.

## 2. General System Architecture

**Figure 1.**
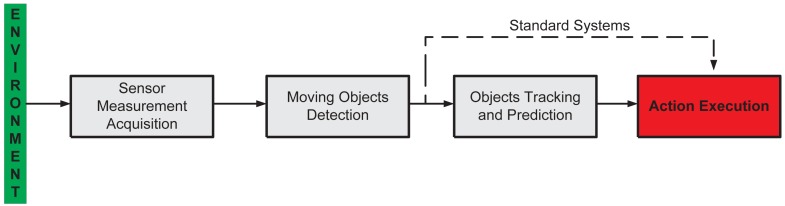
General system architecture.

Figure 1 shows the general system architecture of the proposed supervision system. It is composed by four stages explained as following:

- *Sensor Measurement Acquisition*. Concerns the sensor functionality and the environment information acquisition. In this work, we use range laser sensors to acquire the information of the surrounding environment.
- *Moving Objects Detection*. The environmental information acquired by the sensors is used to detect the presence of objects—e.g., persons, animals, vehicles, *etc.*—within the sensed workspace.
- *Action Execution*. If the detected moving object falls within the restricted region of the workspace, then the system generates the appropriate action, depending on the task in which the supervision system is applied—for example, alarm activation, machinery emergency stop, *etc.*

The abovementioned three stages form a standard supervision system [31]. In our work, we include an extra stage: *Objects Tracking and Prediction*. Thus, in case where an object is detected within the sensed workspace, this extra stage will allow for the prediction of the movement of such an object. With the prediction information available, the system is able to execute the appropriate action before the object enters the forbidden—or restricted—workspace, protecting in that way both the object's integrity and the functionality of the *main process*.

**Figure 2.**
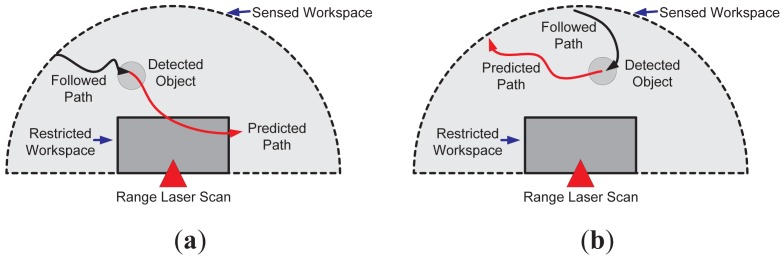
Examples of object prediction. (**a**) shows the case when the predicted movements fall within the restricted region of the workspace; (**b**) shows the case when the prediction does not fall within the restricted area.

It is worth mentioning that such a prediction of the object's movements can be used for the optimization of the sensed workspace by reducing its restricted region. Since the action execution is based on the prediction information, if the predicted object's movements do not compromise the process nor its integrity, then there is no need of an action execution. Nevertheless, the last statement is strongly related to the adopted horizon of prediction. Figure 2 shows an example of this situation. Figure 2(a) shows the case when the predicted movement (solid red arrow) enters the restricted region of the workspace (solid grey), whereas Figure 2(b) shows the case when the predicted object's movements do not trespass the forbidden workspace. In both cases, a range laser sensor was used to depict the examples.

In the following sections, each stage of Figure 1 will be explained in detail. However, as stated in Section 1, this work is focused on the *Objects Tracking and Prediction* stage.

### 2.1. Sensor Measurement Acquisition

**Figure 3.**
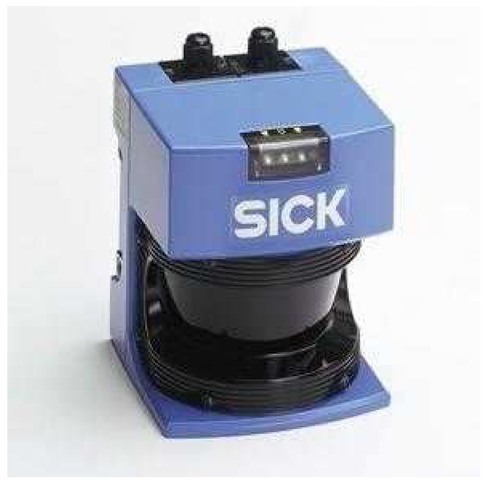
Range laser sensor used in this work.

In this work, *SICK* range laser sensors were used, as the one shown in Figure 3. Such sensors acquire 181 range measurements from 0 to 180 degrees up to a range of 30 meters. As will be shown later, several of these sensors were used during the experimentation. Although in this work range laser measurements are processed, the mathematical formulation of our proposal is not restricted to the nature of the sensor used. Therefore, other sensors such as artificial vision systems, ultrasonic sensors or TOF cameras can be used instead.

### 2.2. Restricted Region Determination

**Figure 4.**
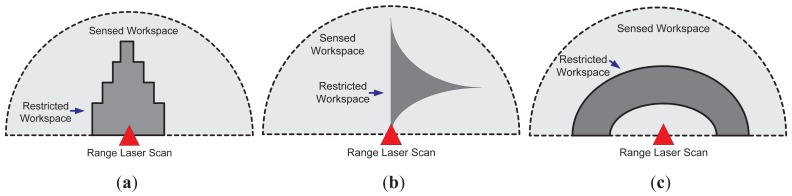
Three examples of restricted workspace configuration.

The restricted workspace determination, as shown in Figure 2, is based on the supervision application. Figure 4 shows three different cases; Figure 4(a) shows the case where a symmetric restricted region is used (solid dark grey). Such a case can be useful in approaching alert situations. Figure 4(b) shows an asymmetric restricted region (also in solid dark grey); such a situation is useful when a non-conventional region of the workspace needs to be supervised. On the other hand, Figure 4(c) shows the case of a restricted workspace suitable for robot manipulator implementations, as the one shown in [35]. It is worth mentioning that the restricted workspace determination is a designer criterion. In addition, two or more laser sensors can be used for defining the restricted workspace, as will be shown in Section 3.

### 2.3. Object Detection

In this work, the detection of moving objects within the sensed workspace shown in Figures 2 and 4 is based on point-based features detection previously presented in [3,4]. Briefly, such a method can be described as follows:

- From the set of 181 measurements acquired by the range laser sensor, the histogram method [15] is used to determine possible point-based features and their corresponding covariance matrices.
- If two or more consecutive measurements are associated to a same point-based feature, then its *center of mass* is determined.
- Each *center of mass* of the detected features is composed by three parameters: its range, angle and covariance matrix. The range is the distance from the *center of mass* to the laser position; the angle is the orientation of the *center of mass* with respect to the orientation of the laser; the covariance matrix is the variance associated with the detection method.
- The parameters of each detected feature are transformed according to a global Cartesian reference frame attached to the system (*x_i_* and *y_i_*, where *i* stands for the *i^th^* detected feature).
- If the same object is detected in two consecutive laser scans, then we are able to track it. In order to do so, a matching criterion must be adopted; *i.e.*, the object detected in time *t* + 1 should be the same than the one detected in time *t*. The Mahalanobis distance [16] was used in this work to match detected features.

It is worth mentioning that the object detection method mentioned above allows for the detection of multiple objects. Further information regarding such a method can be found in [3,4].

### 2.4. Prediction and Tracking: Mathematical Formulation

The linear prediction formulation proposed in this work is based on the Taylor's series expansion [2,9]. By using the Taylor's series, we are able to predict the motion associated with the detected moving obstacles in the workspace of the sensor. In order to illustrate our proposal, let us suppose the following: let *x*(*t*) be the instantaneous position of a body moving along the *x* coordinate in Equation (1) (with constant acceleration). Thus,

(1) $$x(t)=x(t_{0})+v(t_{0})(t-t_{0})+\frac{a(t_{0}){\left(t-t_{0}\right)}^{2}}{2!}$$

where *t* represents time, *t*_0_ is the initial instant, *x*(*t*_0_) is the body's initial position, *v*(*t*_0_) is its velocity and *a*(*t*_0_) is its acceleration. The Taylor's expansion of *x*(*t*) is of the form shown in Equation (2).

(2) $$x(t)=x(t_{0})+\frac{dx(t_{0})}{dt}(t-t_{0})+\frac{1}{2!}\frac{d^{2}x(t_{0})}{dt^{2}}{\left(t-t_{0}\right)}^{2}+R_{m}$$

In Equation (2), *R_m_* is a residual term which contains the higher order values regarding the Taylor's expansion of *x*(*t*). If we compare Equation (1) to Equation (2), we can see that both expressions match and that we can use the Taylor's expansion to estimate the motion of a given object by discarding *R_m_*. In fact, the horizon of our estimation is associated with *R_m_* due to the following:

- In order to estimate *x*(*t*) by using the Taylor's series expansion shown in Equation (2), then *x*(.) belongs at least to *C*^2^, where *C*^2^ is the space of continuous functions with first and second differential also continuous.
- If *x*(.) ∈ *C*^3^, then Equation (2) may include a term from *R_m_* associated with the third differential of *x*(*t*). Thus, the horizon of prediction is increased.
- In general, if *x*(.) ∈ *C^n^*, then the Taylor's expansion of *x*(*t*) can be up to its *n^th^*–differential term.

In addition, if we consider the Euler approximation: $\frac{dx(t)}{dt}\approx \frac{x(t_{x})-x(t_{k-1})}{t_{x}-t_{k-1}}$ for Δ_*t*_ = *t_k_* − *t*_*k*−1_ sufficiently small, we can apply such an approximation to Equation (2) as shown below. Thus, for *x*(.) ∈ *C*^0^:

(3) $$x(t_{k+1})\approx x(t_{k})$$

With the same insight, for *x*(.) ∈ *C*^1^:

(4) $$x(t_{k+1})\approx x(t_{k})+\frac{x(t_{k})-x(t_{k-1})}{t_{k}-t_{k_{1}}}(t_{k}-t_{k_{1}})=2x(t_{x})-x(t_{k-1})$$

In addition, for *x*(.) ∈ *C*^2^ and considering that Δ*_t_* = *t_i_* − *t*_*i*−1_ for *i* = 0‥*k* + 1:

(5) $$x(t_{k+1})\approx x(t_{x})+\frac{x(t_{x})-x(t_{k-1})}{\Delta_{t}}(\Delta_{t})+\frac{1}{2!}\frac{\left(x(t_{k})-x(t_{k-1}))-(x(t_{k-1})-x(t_{k-2})\right)}{\Delta_{t}^{2}}(\Delta_{t}^{2})=\frac{5}{2}x(t_{k})-2x(t_{k-1})+\frac{x(t_{k-2})}{2}$$

Therefore, if the sampling time Δ*_t_* is constant, we are able to find a prediction of *x*(*t*) for *x*(*t*_*k*+1_) based on the Taylor's series expansion. The extension of the procedure shown in Equations (3)–(5) for *x*(.) ∈ *C^n^* is straightforward.

For the multi-dimensional case, let *f*(*t*) be an *b*-dimensional function such that *f*(*t*) ∈ *R^b^*—where *R* is the space of the real valued numbers—and that *f*(.) ∈ *C^l^*. Thus, the Taylor's series expansion of *f*(*t*) is of the form:

(6) $$f(t)=\sum_{p=0}^{l}\frac{\Delta^{p}(f(t_{k}))}{p!}{\left(t-t_{k}\right)}^{p}+R_{m}$$

In Equation (6), *f* is expanded around *t_k_* and Δ*^p^*(*f*(*t_k_*)) is the *p^th^* differentiation of *f* with respect to *t* around *t_k_*. By applying the procedure shown in Equations (3)–(5) and taking into account that Δ*t* = *t_i_* − *t*_*i*−1_ for *i* = 1…*k* + 1, we have that, for the three cases (*f*(.) ∈ *C*^0^, *f*(.) ∈ *C*^1^ and *f*(.) ∈ *C*^2^):

(7) $$\{\begin{array}{l} f(t_{k+1})\approx f(t_{k})\\ f(t_{k+1})\approx 2f(t_{k})-f(t_{k-1})\\ f(t_{k+1})\approx \frac{5}{2}f(t_{k})-2f(t_{k-1})+\frac{f(t_{k-2})}{2} \end{array}$$

Furthermore, for the two-dimensional case (*i.e., f*(*t*) ∈ *R*^2^) and taking into account the object detection procedure presented in Section 2.3, let [*x_i,t_k__ y_i,t_k__*]*^T^* be the coordinates of the *i^th^* detected object at time *t_k_*, with respect to a global Cartesian reference frame. Then,

(8) $$\left[\begin{array}{l} x(i,t_{k+1})\\ y(i,t_{k+1}) \end{array}\right]=\frac{5}{2}\;\left[\begin{array}{l} x(i,t_{k})\\ y(i,t_{k}) \end{array}\right]-2\;\left[\begin{array}{l} x(i,t_{k-1})\\ y(i,t_{k-1}) \end{array}\right]+\frac{1}{2}\;\left[\begin{array}{l} x(i,t_{k-2})\\ y(i,t_{k-2}) \end{array}\right]+R_{m}$$

where *R_m_* ∈ *R*^2^. If we consider that the motion of the detected object falls within *C*^2^, then Equation (8) offers a suitable solution for predicting the motion of the object (*R_m_* should be discarded). In addition, given the algebraic formulation of the proposal, such a predictive approach can be implemented embedded in both low cost and high cost micro-controllers.

It is worth mentioning that, if more precision is required, the number of terms in Equation (8) should be extended (e.g., up to its *n^th^* term). Equation (8) is the one implemented in this work for the motion prediction of the detected objects, because it considers the velocity and the acceleration (associated with the inertia) of the object (see Equation (1)). In addition, Equation (8) can be applied to human motion and to mobile robot's motion [28,30].

By inspection we can see that, if *f*(.) ∈ *C*^2^, then we need the previous knowledge of *f*(*t_k_*_−1_) and *f*(*t_k_*_−2_) in order to predict *f*(*t_k_*_+1_). Therefore, the very first prediction of the process should consider *f*(*t_k_*_−1_) and *f*(*t_k_*_−2_) as a previously defined values (e.g., zero). In our implementations, due to the errors associated with the first predictions, we have discarded the first two predictions.

In addition, if an *r* times forward prediction is expected after one object detection (at time *t_k_*), then the expression in Equation (8) can be successively applied to obtain a prediction up to time *t_k_*_+_*_r_*.

### 2.5. Action Execution

The action execution, as shown in Figure 1, is a designer criterion and it is strictly related to the supervision application nature. Depending on the application, the following situations might apply:

1. *Surveillance*. For stationary lasers disposition, a supervision application can be used to predict the presence of intruders. In such a case, an alarm activation can be used as an *action* once the intruder's trespass have been predicted.
2. *Risk management*. The supervision system can be used to detect when a worker is near a dangerous place within the factory—such as automobile assembly lines, in which robot manipulators are in charge of the mechanic work. Thus, for example, once the presence of a worker within the restricted workspace is predicted, the productive process can be stopped until the risk to the worker's integrity is no longer present.
3. *Vehicles navigation*. For autonomous vehicle navigation, a supervision application can be used for reactive behavior under non-expected situations, such as avoiding obstacles, emergency stops, tangential deviation, among others [4,16,36].

Although several actions can be taken into account according to the application requirements, this work is focused on the *Objects Tracking and Prediction* stage, as stated in Section 2.

## 3. Experimental Results

Several experimental results were carried out in order to show the performance of the proposal. They can be grouped as follows:

- Single laser with single object prediction.
- Single laser with multiple objects prediction.
- Multiple lasers with single object prediction.
- Multiple lasers with multiple objects prediction.

For each mentioned case, 50 trials were run for two different restricted workspace dispositions, see Figure 2. In 25 trials, the intention of the object was to trespass the restricted workspace, whereas in the remaining 25 trials, the intention was the opposite. Up to three persons were considered as moving objects for our supervision application. Each trial consisted of a different path followed by the subjects. In addition, a second order prediction model (see Equation (8)) was associated with the subjects' motion; *r*, the forward time of prediction, was set to *r* = 10 and *r* = 50 (thus, we are able to predict up to *t_k_*_+_*_r_*, as previously mentioned). Considering that the sampling time of the system was set to Δ*_t_* = 0.1 seconds, then with *r* = 10 and *r* = 50 we are able to predict the motion of the objects up to one and five seconds forward, respectively, in the same trial. However, this value can be changed depending on the application's requirements and the object's behavior. The statistical results presented below for each mentioned case show the precision of our proposal to predict trespassing situations.

### 3.1. Single Laser with Single Object Prediction

**Figure 5.**
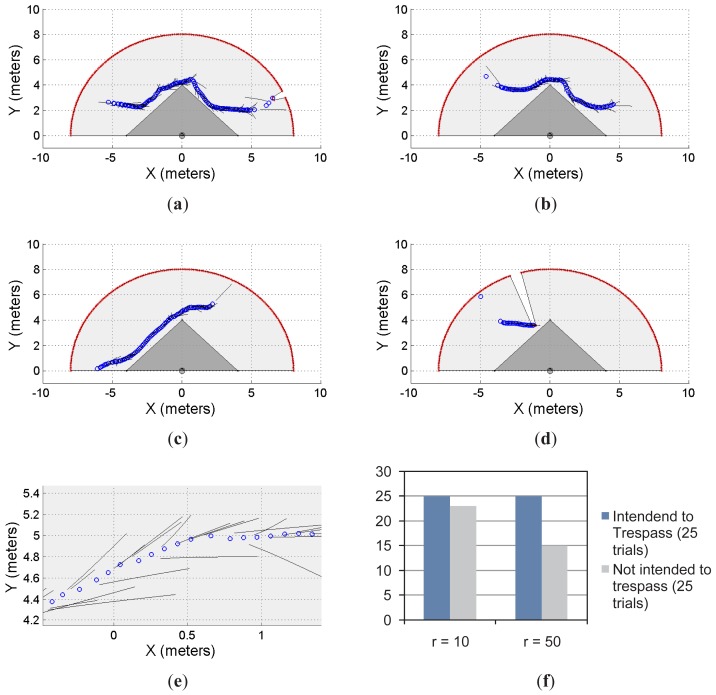
Single object prediction approach: first case. (**a–d**) different cases; (**e**) a close-up of the predicted movements; (**f**) the statistical results of the experimentation.

**Figure 6.**
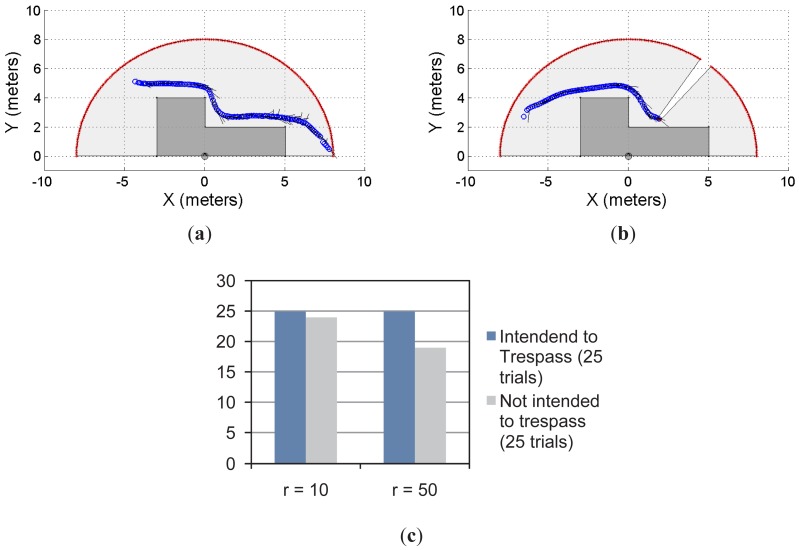
Single object prediction approach: second case. (**a,b**) two examples; (**c**) the statistical results of the experiment.

Figures 5 and 6 show two different restricted workspaces (solid dark grey). The range laser measurements are represented by red dots and the scanned area is in light grey. The blue circles represent the estimated object's position. Such an estimation is performed by the object detection procedure presented in Section 2.3. For visualization purposes, the Cartesian coordinate frame is attached to the sensor's position ([*x_laser_ y_laser_*]*^T^* = [0 0]*^T^*, with an orientation *θ_laser_* = *π*/2) and the detected objects are referred to such a coordinate frame. The small black segments associated with the estimated objects (blue circles) represent the path predicted by our proposal. Such a path is based on the successive prediction of the object's position made by the Taylor's series expansion, as previously shown in Equation (8).

Figure 5(a)–5(d) show four different situations in which our proposal predicts the single object movements; Figure 5(e) shows a close-up of Figure 5(c) for visualization purposes of the prediction behavior. Figure 5(f) shows the statistical results for this single object first approach. With *r* = 10 and for 25 trials in which the object/subject was intended to enter into the restricted workspace, our proposal was able to predict 100% of the cases of such a trespassing intention. However, for 25 trials in which the object/subject was not intended to trespass, our system was able to detect only 92% of the cases (*i.e.*, 23 trials) of such an intention of not trespassing. As can be seen, we have obtained a high rate of positive predictions.

In addition, with *r* = 50 and for 25 trials in which the object/subject was intended to trespass, our system was able to predict the 100% of the cases. However, for 25 trials in which the object/subject was not intended to trespass, we were able to predict the 60% of the cases (*i.e.*, 15 trials). That is, in the 40% of the remaining trials our system predicted the subject's intention (using Equation (8)) to be to trespass when his/her actual intention was the opposite. Such a 60% prediction correctness is due to the horizon of prediction (*r* = 50). With *r* = 10 our system was able to predict the subject's motion up to one second before the motion; however, for *r* = 50, our proposal predicts the subject's behavior up to five seconds before his/her movements. Therefore, a higher rate of false predictions was expected.

Figure 6 shows another example of the single object prediction for a single-laser supervision application. Figure 6(a) and 6(b) show two trials. The restricted workspace is different from the one shown in Figure 5. For this new scenario, the statistical results, presented in Figure 6(c), show that for *r* = 10 and *r* = 50, the system was able to predict the 100% of the cases when the object/subject was intended to enter into the restricted workspace. However, when the intention of the object/subject was to not trespass, the proposed system was able to detect such an intention for 96% of the cases with *r* = 10 and for 76% of the cases with *r* = 50. As can be seen, the statistical results shown in Figure 5(c) shows the same behavior than the results shown in Figure 5(f). Nevertheless, the results shown in Figure 6 are slightly better than the ones shown in Figure 5.

### 3.2. Single Laser with Multiple Objects Prediction

**Figure 7.**
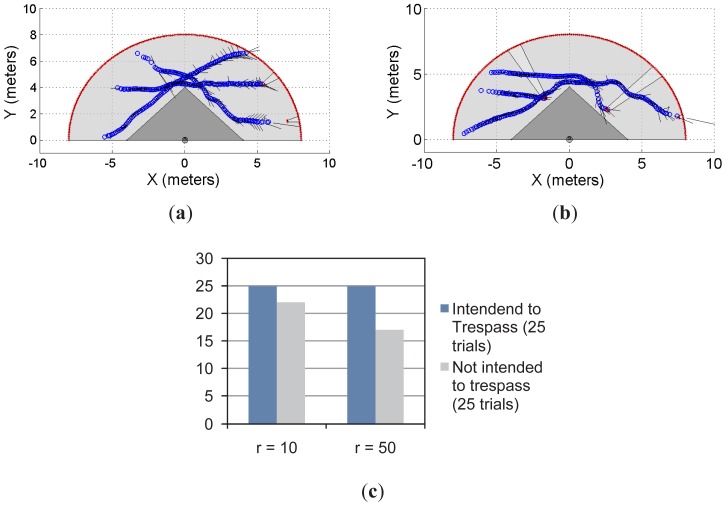
First approach of the multi-object detection using a single laser sensor. (**a,b**) two examples; (**c**) the statistical results of the experiment.

**Figure 8.**
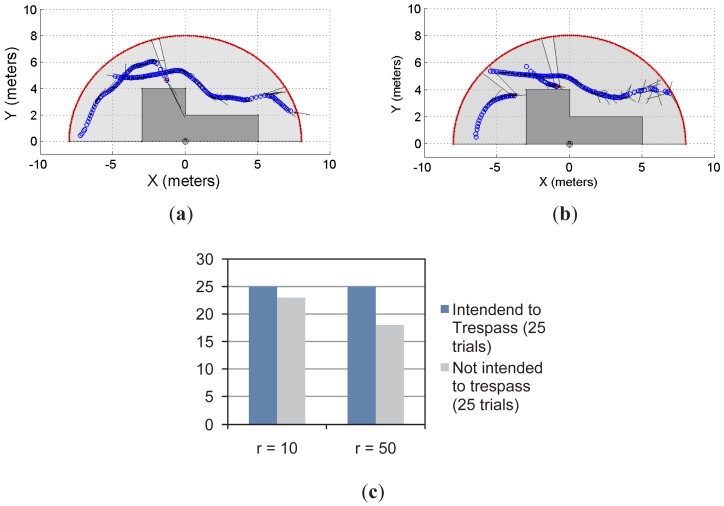
Second approach of the multi-object detection using a single laser sensor.

Figures 7 and 8 show the multi-objects case for the restricted workspaces shown in Figures 5 and 6, respectively. In both cases, up to three subjects/objects were detected.

In Figure 7(a), the object/subject's intention was to avoid the restricted workspace whereas in Figure 7(b) at least one subject intends to trespass such a workspace. Figure 7(c) shows the statistical results from 50 trials; in the first 25 trials, at least one of the object/subject's intention was to enter into the restricted workspace. In the remaining 25 trials, the intention of the moving objects was to avoid trespassing. As can be seen, with *r* = 10 and *r* = 50, our system has predicted 100% of trespassing cases. However, for the prediction of the not trespassing case, our proposal presented a 92% of success for *r* = 10 and 72% for *r* = 50.

With the same insight, Figure 8 shows two examples of the multi-object detection using the restricted workspace shown in Figures 6–8(b). In addition, Figure 8(c) shows the statistical results for the experiment. As can be seen, for *r* = 10 and *r* = 50, there is 100% of achievement when the system is used to predict the trespassing of multiple objects when they intended to do so. However, when the intention was to avoid trespassing, for *r* = 10 the system showed 92% of effectiveness; for *r* = 50, the system showed 72% of effectiveness in the prediction. It is worth mentioning that, as stated for the previous experiment, 25 trials were run for each situation shown in Figure 8(c).

### 3.3. Multiple Lasers with Single Object Prediction

**Figure 9.**
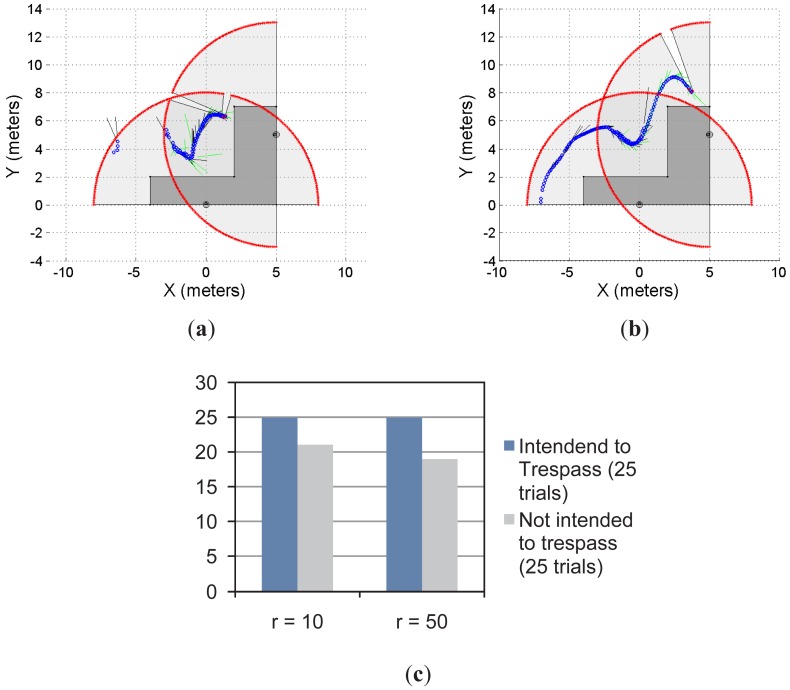
Single object prediction by a multi-laser disposition. (**a,b**) two examples of the performed experiment; (**c**) the statistical results of the experiment.

**Figure 10.**
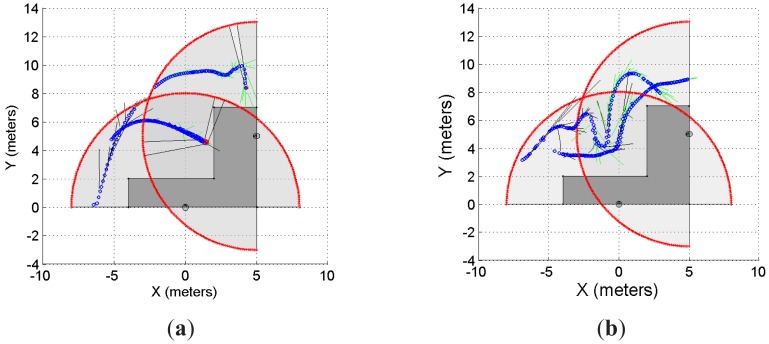

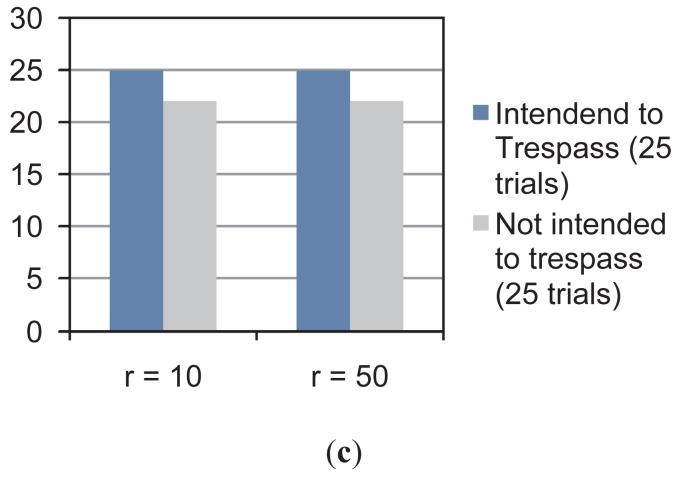
Multi-object prediction using a multi-laser disposition. (**a,b**) two examples of the performed experiment; (**c**) the statistical results of the experiment.

As previously stated, we have implemented our supervision application to a system with multiple range lasers, as the one shown in Figures 9 and 10. The sensors disposition is as follows: one laser is located at [*x*_*laser*,1_ *y*_*laser*,1_]*^T^* = [0 0]*^T^*, with an orientation *θ*_*laser*,1_ = *π*/2; whereas the second laser is located at [*x*_*laser*,2_ *y*_*laser*,2_]*^T^* = [5 5]*^T^*, with an orientation *θ*_*laser*,2_ = *π*. The maximum range of measurement, for both lasers, is set to 8 meters. The solid grey area is the common restricted workspace. The first laser prediction is drawn in solid black segments whereas the second laser prediction is drawn in solid green segments. It is worth mentioning that each laser has implemented the prediction strategy proposed in this work; in addition, they work independently. The latter means that each laser has associated its own predictor based on its own moving object detection stage. Thus, one laser might detect the moving object in a different position than the detection performed by the other laser. This is so because the histogram detection method used herein ([15]) depends on the shape of the object. Then, if two detections are different, their corresponding predictions might be different as well, as shown in Equation (8). Blue circles in Figures 9 and 10 represent the detected object.

Figure 9(a) and 9(b) show two examples of the experiment carried out using two lasers and a common restricted workspace; Figure 9(c) presents the statistical results of the experiment which are consistent with the results shown for the previous experiments. For *r* = 10 and *r* = 50, the system predicted the 100% of the cases when the intention of the object/subject was to trespass the restricted workspace. However, when the intention was to avoid trespassing, the system predicted the 84% of the cases when *r* = 10 and 76% when *r* = 50. It is worth mentioning that 25 trials were carried out for each case, as stated in the previous sections.

### 3.4. Multiple Lasers with Multiple Objects Prediction

As mentioned in Section 3.3, Figure 10 shows the multi-object prediction by a multi-laser system using a common restricted workspace. Up to three objects/subjects were part of the experiment. Figure 10(a) and 10(b) show two examples of this situation whereas Figure 10(c) shows the statistical results. As can be seen, when the intention was to enter into the restricted workspace, the system was able to predict the 100% of the cases for both *r* = 10 and *r* = 50. However, as observed for the previous experiments, when the intention was to avoid trespassing, for *r* = 10 the system showed 88% effectiveness. For *r* = 50, the system showed also 88% effectiveness.

## 4. Discussion and Lessons Learned

During the experimentation, a number of lessons were learned with regard to the supervision application strategy based on the Taylor's prediction criterion proposed in this work. First, the precision of the prediction is strongly related to the precision of the detection procedure. Thus, a noisy detection procedure can transform a movement that originally belongs to *C*^2^—which is associated with a smooth movement—into a movement that belongs to *C*^0^, such as an arbitrary movement.

Second, the effectiveness of the prediction is also affected by the horizon used in the implementation. As shown in the statistical results in Sections 3.1–3.4, for *r* = 10 we have obtained better results in predicting the motion of the objects when the intention was to avoid the restricted workspace. However, in general, for *r* = 50 the system has shown worst results, mainly due to the fact that when *r* = 50 the system formulates a prediction up to 5 seconds forward. As expected, during this time, the object can change its motion which can make the prediction to fail. It is worth mentioning that, once the predicted motion had estimated that the object will trespass, the action execution of the system was to emit an alarm.

Third, the multi-object case with both single or multi-laser situations has shown similar results than the single-object case. Despite the fact that each object was predicted independently, the objects detection procedure used has shown to be efficient for the application. In addition, it is worth mentioning that we have used subjects as objects for the execution of the experimentation.

Fourth, if the detected object moves following a straight line and the detection method is too noisy, then the system will face a situation in which a linear movement might be detected as a random one. Considering that the error in the detection is propagated to the prediction, it also conditions the order of the Taylor's expansion prediction to be used in the process. Therefore, it is recommendable to have previous information regarding the detection method's efficiency before choosing the order of the Taylor's expansion and its corresponding horizon of prediction.

## 5. Conclusions

This paper has presented a new prediction method based on the Taylor's series expansion of the motion of an object given its detection parameters. The accuracy of the method is related to the maximum order adopted for the Taylor's expansion. Considering the algebraic formulation of the prediction method, it is suitable for implementation in embedded systems. Also, it is scalable: it can be adapted to the number of objects whose motions are going to be predicted.

In addition, the proposed method was implemented in range laser-based supervision systems for the prediction of trespassing situations. Such situations are common when working in restricted environments and free motion is not allowed due to risky situations. Our proposal has shown to be effective to predict future trespassing situations. With this insight, two main cases were presented: the single-laser case with both single object and multi-object prediction; and the multi-laser case with both single object and multi-object prediction. For all the cases, our proposal had shown a 100% of effectiveness in predicting intended trespassing situations. However, the system had also predicted false trespassing situations—*i.e.*, the object moved close to the restricted workspace without trespassing it. It is worth mentioning than 25 trials were run for each experimental case using two different horizon values: the prediction up to 10 times forward and up to 50 times forward. Predictions with an horizon of 10 times forward have shown better statistical results than predictions with an horizon of 50 times forwards. Several workspace dispositions were used to test our proposal.
